# Development of Physiologically Based Pharmacokinetic (PBPK) Modeling

**DOI:** 10.3390/pharmaceutics18020238

**Published:** 2026-02-13

**Authors:** Bárbara Sánchez-Dengra, Isabel González-Álvarez

**Affiliations:** Engineering, Pharmacokinetics and Pharmaceutical Technology Area, Miguel Hernandez University, 03550 San Juan de Alicante, Alicante, Spain

In this Special Issue, “Development of Physiologically Based Pharmacokinetic (PBPK) Modeling”, readers can find a selection of articles that discuss the most current challenges and advances in this area.

PBPK modeling is a mathematical tool which is able to describe the drug concentration in an organism by using compartments and taking into account the physiology of this organism [1].

Specifically, this Special Issue comprises ten articles (two review articles and eight research articles) and illustrates how PBPK has evolved from a theoretical tool to an indispensable mechanistic and predictive platform. Through reading these articles, readers will realize that PBPK is able to help in critical decision-making, such as dose optimization in vulnerable populations, but also in analyzing drug interactions, virtual bioequivalence and the efficacy of complex formulations.

## 1. PBPK Modeling in Special Populations

In their review article, Gonnabathula et al. investigate how PBPK modeling can be used to assess drug metabolism and other ADME processes within particular demographic groups. For instance, they highlight the importance of considering physiology genetics, developmental stage and health status as key factors influencing pharmacokinetics. In addition, they conclude that recognizing and integrating these factors into PBPK modeling supports dose selection, improves the results of clinical trials and strengthens regulatory applications (contribution 1).

Drug metabolism is profoundly influenced by genetic factors, particularly polymorphisms in cytochrome P450 enzymes and differences in allele frequencies between ethnic groups [2,3]. In addition, the ADME processes are not static and change drastically throughout a person’s life, and pathological conditions alter physiology in ways that significantly impact pharmacokinetics; for example, renal and hepatic insufficiency directly reduce elimination, while obesity alters the volume of distribution and enzyme activity [4]. Gonnabathula and collaborators show that PBPK models can simulate such conditions by adjusting key parameters such hepatic blood flow, organ-specific enzyme activity or body composition to predict drug exposure in patients. This is especially notable in populations where clinical studies are limited or present ethical dilemmas, such as in pediatric, geriatric or pregnant women, allowing for informed extrapolation from healthy adult data (contribution 1).

The research articles by Costa et al. and Liu et al., also included in this Special Issue, are examples where physiological changes drastically alter the pharmacokinetics of drugs such as efavirenz, lamotrigine and meropenem in pregnant women and critically ill children (contributions 2 and 3).

In their work, Costa et al. report two PBPK models for pregnant women with differing performance: the model for lamotrigine successfully reflects the in vivo outcomes, whereas the efavirenz model does not capture them accurately. In the case of lamotrigine, simulation of a constant dose of 200 mg shows a progressive decrease in drug exposure as pregnancy progresses, reflecting the increased metabolic clearance that can be explained by inducing metabolic enzymes such as UGT during pregnancy [5]. The efavirenz model, however, significantly underestimates both C_max_ and AUC during pregnancy, probably due to genetic variability. Despite not being completely successful, this project contributes to the larger goal of treating expectant mothers with safe and efficient treatments, improving the health of not only the mothers but also their babies (contribution 2).

Liu et al. worked with critically ill children with kidney function significantly above normal levels for their age who were treated with the antibiotic meropenem. These children suffer hyperfiltration and standard doses usually lead to subtherapeutic concentrations, compromising treatment efficacy and increasing the risk of therapeutic failure and bacterial resistance. The study used two complementary modeling approaches to analyze the pharmacokinetics of meropenem (PopPK and PBPK). The PopPK model showed better predictive ability, especially in subgroups with a high estimated glomerular filtration rate (eGFR), while the PBPK model displayed higher precision in the low-eGFR subgroup. The main conclusion of the study, supported by both models, was unequivocally that standard doses of meropenem are insufficient for critically ill pediatric patients with augmented renal clearance (contribution 3).

In addition to guiding dosing in special populations, PBPK modeling is a vital tool for unraveling complex pharmacokinetic mechanisms, and this is also shown in this Special Issue.

## 2. Mechanistic Analysis of Pharmacokinetic Phenomena

In Slavsky et al.’s publication, PBPK modeling is used to study drug–drug interactions (DDIs). Specifically, they research the induction of CYP2C metabolic enzymes, whose study with traditional models, such as mechanistic static modeling (MSM), often fails because of the low magnitude of in vitro CYP2C induction in comparison with that of CYP3A4, and because many CYP2C substrates are also metabolized by other enzymes [6]. The authors analyze the interactions among different drugs and CYP2C in a hepatocyte triculture and then include the obtained data in a PBPK model. This approach demonstrates the superiority of the PBPK approach, which, unlike the MSM, which frequently overestimates or underestimates risk, accurately predicts DDI clinical outcomes (contribution 4).

Drug recycling is a process involving the deconjugation of previously metabolized drugs which can significantly increase systemic exposure and, if it occurs in the gut, can enhance local exposure in intestinal tissue [7]. In the article published by Ramisetty et al., this recycling process is studied for the drug genistein. Normally, genistein is eliminated after being metabolized into glucuronide metabolites. However, gut microbial β-glucuronidase (gmGUS) can reverse this reaction, releasing the original drug for reabsorption. This fundamental role is demonstrated by PBPK modeling, and it is proven that taking this role into account is critical for drugs targeting the colon, since efficacy and toxicity may be driven by local concentrations that are greater than those in plasma (contribution 5).

## 3. Prediction of Exposure at the Site of Action

Ramisetty’s model explains not only the drug recycling phenomena, but also exposure of colonocytes to genistein (contribution 5). This article, in combination with the next four, shows how PBPK modeling can be applied to different therapeutic areas to predict the exposure of a drug at the site of action.

Matharroo and collaborators applied PBPK modeling to predict the performance of three different gel formulations loaded with adapalene after topical administration. The in vitro studies were developed to feed the model, and the simulations confirmed that there were significant differences in drug release and permeation rates depending on the formulation. In addition, a clinical study was developed to link the local concentrations with the efficacy of the formulations. The results showed that different gels produced different effects on the skin, providing a mechanistic view of how small changes in gel formulation impact both the drug delivery to the skin and the final physiological effect. Thus, PBPK tools may assist in refining topical formulations, minimizing the need for in vivo testing and hastening the development of more effective dermatological treatments (contribution 6).

Oncology is another area in which PBPK modeling can be used to predict the concentration of a chemotherapeutic agent directly in tumor tissue. Peribañez-Dominguez et al. and Susilo et al. have published articles using PBPK models to attempt to achieve this purpose (contributions 7 and 8). The objective of the first study was to build PBPK models for four key drugs used in colorectal cancer chemotherapy (5-fluorouracil, leucovorin, oxaliplatin and irinotecan). Tissue distribution analysis revealed that irinotecan showed significantly greater accumulation in colonic tissue compared to plasma. Based on the results obtained in colon studies, it was concluded that the covariates to consider when personalizing dose would be weight and sex (contribution 7). In the second study, thanks to a PBPK model for optimizing the biodistribution of immunotherapy, it was shown that the efficacy of TCB antibodies was affected not only by the dose used but also by the CD3 affinity (contribution 8). On the other hand, the work by Mehta et al. focused on the use of these models to predict the passage of substances across the blood–brain barrier (contribution 9). To do this, they needed to incorporate data obtained from a rat animal model and extrapolate it to humans to successfully predict drug passage into the CNS. All of these studies conclude that PBPK models determine the variables to guarantee therapeutic success.

## 4. PBPK Modeling and Virtual Bioequivalence

The systematic review conducted by Alotaiq and Dermawan highlights the transformation of generic development through the inclusion of computational methods, such as PBPK modeling. This transformation has been promoted by regulatory agencies, such as EMA and FDA, which encourage the use of PBPK modeling to demonstrate bioequivalence, reducing the need for costly and ethically sensitive clinical trials [8,9]. A problem arises from the lack of standardization in these studies, leading to significant variability in published results. Therefore, the incorporation of standard regulations into virtual bioequivalence methods is crucial to guarantee the safety, reproducibility and success of these tools (contribution 10).

All the articles compiled in this Special Issue conclude that computational tools such as PBPK models are indispensable in drug development today. These models are suitable from a mechanistic and predictive perspective and also allow for the extrapolation of results to vulnerable populations; the detection of simple and complex interactions or metabolic processes; and the quantification of drugs in tissues such as the brain, skin or tumors.

Therefore, nowadays, the incorporation of PBPK models is promoted to achieve an advancement towards precision medicine, positioning PBPK modeling not only as a simulation exercise but as an important tool in drug development.
